# Orofacial Migraine and Other Idiopathic Non-Dental Facial Pain Syndromes: A Clinical Survey of a Social Orofacial Patient Group

**DOI:** 10.3390/ijerph20206946

**Published:** 2023-10-20

**Authors:** Federica Reina, Giuseppe Salemi, Mariarita Capizzi, Salvatore Lo Cascio, Antonio Marino, Giuseppe Santangelo, Andrea Santangelo, Mirko Mineri, Filippo Brighina, Vincenzo Raieli, Carmelo Attilio Costa

**Affiliations:** 1 Child Neuropsychiatry Unit Department, Pro.M.I.S.E. “G D’Alessandro”, University of Palermo, 90100 Palermo, Italy; federica.reina@you.unipa.it (F.R.); mariarita.capizzi@community.unipa.it (M.C.); salvatore.locascio02@community.unipa.it (S.L.C.); antoniomarino94@tiscali.it (A.M.); 2 Department of Experimental Biomedicine and Clinical Neurosciences, University of Palermo, 90100 Palermo, Italy; giuseppe.salemi@unipa.it (G.S.); filippo.brighina@unipa.it (F.B.); 3 Child Neuropsychiatry Department, ISMEP—ARNAS Civico Palermo, via dei Benedettini 1, 90100 Palermo, Italy; giuseppe.santangelo@arnascivico.it; 4 Pediatric Neurology, Pediatric Department, AOUP Santa Chiara Hospital, 56121 Pisa, Italy; androsantangelo@gmail.com; 5 Pain Management Department, Humanitas, 95045 Catania, Italy; mirko.mineri@humanitascatania.it (M.M.); carmelo.costa@humanitascatania.it (C.A.C.)

**Keywords:** orofacial pain syndrome, non-dental facial pain, headache, migraine, trigeminal neuralgia, cluster headache

## Abstract

**Background**: Orofacial pain syndromes (OFPs) are a heterogeneous group of syndromes mainly characterized by painful attacks localized in facial and oral structures. According to the International Classification of Orofacial Pain (ICOP), the last three groups (non-dental facial pain, NDFP) are cranial neuralgias, facial pain syndromes resembling primary headache syndromes, and idiopathic orofacial pain. These are often clinical challenges because the symptoms may be similar or common among different disorders. The diagnostic efforts often induce a complex diagnostic algorithm and lead to several imaging studies or specialized tests, which are not always necessary. The aim of this study was to describe the encountered difficulties by these patients during the diagnostic–therapeutic course. **Methods**: This study was based on the responses to a survey questionnaire, administered to an Italian Facebook Orofacial Patient Group, searching for pain characteristics and diagnostic–therapeutic care courses. The questionnaire was filled out by patients affected by orofacial pain, who were 18 years and older, using a free online tool available on tablets, smartphones, and computers. **Results**: The sample was composed of 320 subjects (244F/76M), subdivided by age range (18–35 ys: 17.2%; 36–55 ys: 55.0%; >55 ys 27.8%). Most of the patients were affected by OFP for more than 3 years The sample presented one OFP diagnosis in 60% of cases, more than one in 36.2% of cases, and 3.8% not classified. Trigeminal neuralgia is more represented, followed by cluster headaches and migraines. About 70% had no pain remission, showing persisting background pain (VAS median = 7); autonomic cranial signs during a pain attack ranged between 45 and 65%. About 70% of the subjects consulted at least two different specialists. Almost all received drug treatment, about 25% received four to nine drug treatments, 40% remained unsatisfied, and almost 50% received no pharmacological treatment, together with drug therapy. **Conclusion**: To the authors’ knowledge, this is the first study on an OFP population not selected by a third-level specialized center. The authors believe this represents a realistic perspective of what orofacial pain subjects suffer during their diagnostic–therapeutic course and the medical approach often results in unsatisfactory outcomes.

## 1. Introduction

Orofacial pain syndromes (OFPs) are a heterogeneous group of syndromes mainly characterized by painful attacks localized in facial and oral structures [1]. The International Classification of Orofacial Pain (ICOP) [1] divided OFPs into six major groups. The first three are disorders of dentoalveolar and anatomically related structures, myofascial facial structures, and the temporomandibular joint. The remaining three groups (non-dental facial pain, NDFP) are cranial neuralgias, facial pain syndromes resembling primary headache syndromes, where the pain is located below the orbitomeatal line [2], and idiopathic orofacial pain. Trigeminal neuralgia is a disorder characterized by severe short-lasting, lancinating pain, without cranial autonomic symptoms. It can be triggered by triggers and is easily recognized if known. The family doctor should send the patient to a neurologist who can initiate specific neuroradiological (MRI) investigations, avoiding unnecessary X-rays. The first line of treatment is with medications; invasive treatment options should be considered only if pharmacotherapy is ineffective. The fifth group is facial pain syndromes that most closely resemble well-known primary headache syndromes, such as migraine, but with pain located below the orbitomeatal line. These syndromes may be treated similarly to the corresponding types of primary headaches. If one is not misled by the atypical site of localization, assessing the recurrence of the attacks, their duration, and the accompanying general and local signs, one can avoid unnecessary examinations and invasive dental maneuvers. Idiopathic facial pain (PIFP) is a chronic pain disorder with persistent pain in the face and/or teeth, often burning, not secondary to other disorders, and without any structural anomalies. This type of pain may chronicize after invasive procedures; therefore, dental procedures should not be performed if the teeth are healthy. The treatments are mainly antidepressant and anticonvulsive drugs [3,4,5,6,7,8]. While the first three groups primarily refer to a dentistry specialist, and the patients frequently find a good diagnostic and clinical response, the last three groups are often a clinical challenge because the symptoms may be very similar or common among the different disorders. Not rarely, all diagnostic attempts can remain unsuccessful without revealing a definite physical intra- or extraoral pain correlation [3,4,5,6,7,8]. For this reason, subjects with OFP often turn to more specialists without obtaining a definitive diagnosis [5]. This could result in the loss of confidence in physicians by patients. Moreover, the persistence of the pain due to a misdiagnosis or ineffective treatment could involve negative psychosocial and functional consequences in multiple areas of the lives of patients [4]. Furthermore, except for trigeminal neuralgia (TN), which is a well-known disorder characterized by recurrent attacks of intense pain along the trigeminal nerve distribution, the other disorders are less studied and less known [5,6]. The diagnostic efforts often lead to a complex diagnostic algorithm and execute several imaging studies or specialized tests, which are not always necessary.

For these reasons, it seems useful to analyze the diagnostic and therapeutic process of a group of NFDP subjects, not selected by clinical services but by a patients’ association, trying to understand not only their clinical features but also the involved diagnostic examinations (also incorrect) and the several adopted therapeutic strategies. Our aim was to describe the difficulties encountered by these patients during the diagnostic–therapeutic process (i.e., delays in diagnosis, useless tests performed) to adopt more effective strategies in the future.

## 2. Materials and Methods

Investigations for this transversal study were carried out by a research group, called GIMDO (Gruppo Italiano Multidisciplinare Dolore Orofaciale), which includes several researchers from different affiliations interested in the study of orofacial pain.

This study was based on the responses to a survey questionnaire administered to an Italian Facebook Orofacial Patient Group (Trigeminal Neuralgia and Orofacial Pain group), aimed to determine pain characteristics and the diagnostic–therapeutic care process. Patients in this social group interact with each other, exchanging advice on specialists, specialized centers, and treatments, and often inviting specialists to discuss with them in webinars. One of the authors CA.C was invited to one of these webinars, so it was possible to establish a collaboration. To be admitted into the social media group, all members had to declare to be affected by TN or other non-dental facial pain. The questionnaire was filled out by patients aged 18 years or older who suffer from orofacial pain using a free online tool on tablets, smartphones, and computers and was conducted between March and April 2021.

The questionnaire was submitted via a link, which was sent by the host of the Facebook group to all members. As a preliminary step, they were asked to give written online consent to the processing of their answers, given anonymously, and to declare that they were over 18 years old; otherwise, the online system would not allow them to continue. Given the descriptive purpose of this study, inclusion criteria were age and being enrolled in the Facebook group, while exclusion criteria were the presence of dental or myofascial syndromes as the first diagnosis and neoplastic diseases. No statistical power calculation was conducted prior to this study, but the sample size was based on the available data.

The sample of potential subjects to be interviewed consisted of 468 subjects active in the Facebook group. They answered the questionnaire in 320 subjects equal to 68.4%.

According to local ethical policies, no formal approval by the hospital ethics committee was needed.

The group of patients was from different regions of Italy. In more detail, participants were divided into four groups. The first group included subjects who affirmed to be affected by TN as their only diagnosis (group A), the second comprised subjects with TN and other comorbidities such as headache, migraine, different neuralgia, etc. (group B), the third comprised subjects with a pathology that caused facial pain without TN, and the fourth included subjects without a specific diagnosis. Overall, the entire examined population was composed of 320 subjects, of whom 76 were male and 244 were female, most of whom were aged between 46 and 55 years.

### 2.1. Survey

The Orofacial Pain GIDMO Survey consisted of a list of 45 questions divided into 7 sections to which all the participants replied using a free online tool. Some of the questions required a yes/no answer and the next one had to be answered only if there was a positive answer to the previous one (close questions). Others were multiple-choice and often, more than one answer was allowed, or an alternative could be added (open questions). In the final part of the questionnaire on the level of pain and satisfaction with the diagnosis, treatments, or degree of side effects, the median and range of the scale used are given. The first section included general subject information such as age, gender, region, and diagnosis. The second and fourth parts focused on pain clinical features, whereas the fifth and sixth parts regarded diagnostic and therapeutic paths. The third and seventh parts were composed of scores ranging from 0 (poor) to 10 (excellent) on a rating system about the acme in pain and intensity of interictal pain, in addition to the degree of treatment and diagnosis satisfaction. The questionnaire was prepared ad hoc. Its administration did not exceed 20–30 min, as evidenced by the administration of some employees.

Two doctors (blinded between them, VR and CC) examined the questionaries using ICOP diagnostic criteria [1] to independently classify the subjects, reallocating them into the four groups. In case of discordance, another doctor (FB) decided the final choice.

### 2.2. Data Analysis

Data analyses were conducted using Microsoft Office Excel, version 16. The median and confidence intervals were used when we were interested in showing measures of central tendency and dispersion of the study population. Percentages were used when we wanted to show the partition of the whole population.

## 3. Results

The sample was composed of 320 subjects (244 women, 76 men). They were divided into age ranges (18–35 ys: 17.2%; 36–55 ys: 55.0%; >55 ys 27.8%). The population was distributed throughout Italy, with 152 subjects from the north, 53 from the center, and 139 from the south and islands, while three patients declared that they lived outside Italy. Most of the patients had been affected by OFP for more than 3 years (Table 1).

The sample showed one OFP diagnosis in 60% of cases, more than one in 36.2% of cases, and 3.8% were not classified. TN was the most represented, followed by cluster headache (CH) and surprisingly, migraine. The absence of persistent idiopathic facial pain (PIFP) was even more surprising. Table 2 shows the distribution of the different diagnoses.

Analyzing the global sample, we observed that most subjects had no remission of pain, interestingly, across the several groups of disorders; in about 30% of cases, the pain showed an intermittent course with free pain remission periods; continuous pain associated with acute exacerbations was prevalent. It was not characterized by sudden onset and end, showing a point median equal to 8/10 on the Visual Analogue Scale (VAS) during acute crisis. The persisting background pain was described with a median of 7 on the VAS point; the background pain was less defined, while the acute pain was predominantly defined as burning or throbbing. Almost all patients did not report important vegetative signs, such as vomiting. The pain was strictly unilateral in 85% of cases. Interestingly, about 65% of the subjects reported pain irradiation beyond the face, such as to the back, neck, ears, etc. Autonomic cranial signs during the pain attack, in oscillating percentages, were present between 45% and 65%, except for facial sweating, which was present in less than 20% of cases. Cutaneous triggers were reported by 61% of patients.

About 40% of the patients identified a triggering cause of pain, such as trauma, herpes infection, surgical and radiotherapy treatment, or especially, dental treatments.

The interview also evaluated which and how many specialists were consulted before and after. Neurologists and dentists were the first to be consulted; however, several specialists were met in various ways. About 70% of the subjects consulted at least one other doctor, and more than 30% at least two others. Frequently, more than one exam was prescribed from the beginning, and about 50% directly underwent a brain or maxillofacial MRI. About 70% of patients received a diagnosis at their first visit, but curiously, about 30% of this group received more than one diagnosis at the first visit. The initial diagnoses did not exactly match the final diagnoses in which the patients were placed, with 26% continuing to have more than one final diagnosis. Overall, 10% still did not have a diagnosis, and finally, there was a group of about 15% of patients whose diagnosis had been matched between 3 and over 5 times.

The last part of the questionnaire was related to the treatment of orofacial pain in this group. Almost all (more than 95%) received drug treatment. About 25% had between four and nine drug treatments prescribed, but 40% remained unsatisfied, and almost half received nonpharmacological treatment, of which about 50% were in association with drug therapy. About 35% of patients received more than one non-pharmacological treatment, and almost 8% received between four and eight non-pharmacological treatments (which are listed in the table). Finally, when patients were asked to rate their degree of satisfaction (both drug therapy and non-drug therapy) on a scale between 0 and 10, the median of responses was 5, apart from the side effects from drug therapies, which had a median response of 8. The reported results are shown in Table 2, Table 3, Table 4, Table 5, Table 6, Table 7 and Table 8.

Upon analyzing the responses, we divided them into three diagnostic groups, and we found that the first group of patients with trigeminal neuralgias had no diagnosis at their first visit in 26.3% of cases. About 80% of these patients consulted multiple specialists, and the diagnoses were changed more than once for about 39% of patients and more than twice for about 20% of patients. Almost all subjects were prescribed pharmacological therapy, and more than 50% were unsatisfied with their first diagnosis and prescribed therapy. About 77% of patients reported discomfort from side effects. Non-pharmacological therapies were used by about 57% of the sample, and about 75% were satisfied with their treatment, but about 58% reported discomfort from these therapies.

The second group included 53 subjects with reported TN together with other orofacial pain syndromes. In this group, 41% had no diagnosis at their first visit, about 50% consulted multiple specialists, and the diagnosis changed more than once for about 67% of patients and more than twice for about 30% of patients or remained without a specific diagnosis. Almost all subjects were prescribed pharmacological therapy (about 92%), and about 68% were unsatisfied with their first diagnosis and prescribed therapy, with more than 70% reporting discomfort from side effects. Non-pharmacological therapies were used by about 57% of the sample, and about 47% were satisfied with the treatment, although about 46% reported discomfort from these therapies.

The third group was grouped with other orofacial pain syndromes not associated with trigeminal neuralgia (131 subjects). We observed that about 30% of patients in this group had no diagnosis at their first visit, and the first-consulted doctor was typically the family physician, in contrast with the other two groups. About 58% consulted multiple specialists, and the diagnosis changed more than once for about 39% of patients and more than twice for 12.1% of patients, or they remained without a specific diagnosis. Almost all subjects were prescribed pharmacological therapy (about 97%), and about 58% were unsatisfied with their first diagnosis and prescribed therapy, with more than 60% reporting discomfort from side effects. Non-pharmacological therapies were used by about 45% of the sample, and about 40% were satisfied with their treatment, but about 44% reported discomfort from these therapies.

The post hoc analysis of the orofacial pain diagnosis was performed by three researchers to evaluate whether the diagnoses assigned by the patients themselves were modified based on their responses to the questionnaire about the reported features of orofacial pain syndromes.

This analysis showed a significant reduction in idiopathic trigeminal neuralgia diagnoses, as more than 40% of them were reclassified as symptomatic trigeminal neuralgias, following mainly dental disorders or dental surgical interventions. The diagnoses of migraine and CH were slightly reduced, and the diagnosis of tension headache was reduced by almost 50%. However, idiopathic persistent facial pain diagnosis remained rare (with only three probable cases).

## 4. Discussion

Facial pain sufferers often go from one specialist to another without finding good relief for their pain [5,6]. These considerations seem to be valid for all orofacial pain pictures, especially in groups 4–6, including disorders such as primary trigeminal neuralgia, which have well-defined diagnostic criteria that should make them easy to diagnose and treat specifically [1,9].

To establish and organize an adequate diagnostic path within the health system, it is essential to know which current diagnostic–therapeutic stages patients suffering from orofacial pain frequently face and whether they are satisfied with the type of attention and care received [4,5,6,7,8]. The collaboration of one of us with a group of patients gathered in an association via social media allowed us to interrogate a large group of subjects, who were not selected for affiliation to a specific specialist center but joined only by the common symptom of orofacial pain, where the diagnostic attribution of trigeminal neuralgia was common. To the authors’ knowledge, this is the first study on a population that was not selected by a third-level specialized center [5,10,11] or on the general population [12] but which spontaneously includes itself in an Association of Trigeminal Neuralgia and Other Orofacial Pains with the diagnosis attributed following visits carried out by the most diverse specialists and in the most diverse locations. This, in our opinion, gives a more realistic depiction of what orofacial pain patients endure during their diagnostic course, as well as how much awareness of their disease they have.

To begin, it is critical to note that, as expected, trigeminal neuralgia is the most common manifestation in our sample, accounting for around 40% of cases. Surprisingly, the sample includes approximately 20% of cases characterized by primary migraine and tension headaches. This incidence far exceeds the rates described in the previous literature, where migraines with prominent facial involvement and tension headaches are considered extremely rare [12].

On the other hand, the recent scientific literature shows that primary headaches can involve the lower half of the skull in about 10–20% of cases [4,5,8,9]. This aspect must be kept in mind because these patients could be mistakenly subjected to invasive interventions, especially dental ones. Furthermore, Ziegler and May [2] recently pointed out that the current upper limit for defining pain as orofacial and not as headache (orbitomeatal line) could easily include many TACs and migraines (trochlear migraine [13,14]) as orofacial pains, and these authors, therefore, suggest a modification of this limit [2]

Even more interesting is the almost complete absence in our sample of persistent idiopathic facial headache (PIF), which accounts for a significant proportion of clinical cases at specialized centers [6,11,15]. There may be several reasons for explaining these conflicting data:

1. The common knowledge of primary headaches among patients and doctors with a preparation not particularly aimed at orofacial pain of non-dental origin can lead to overestimating these diagnoses. In the case of PIFP, its lack of knowledge and the absence of specific characteristics can lead to a strong underestimation, even in a sample of patients selected for orofacial pain to enroll in a specific group [5];
2. It is also possible that patients with PIFP, precisely because of the poorly defined characteristics of their disorder, tend not to join an association to which they think they do not belong. However, this suggests that the patient, though receiving a correct diagnosis, has not received an appropriate elucidation from the referring physician and, therefore, is not able to receive help from these patient associations. The patient probably misses the significance of the disorder, leading the patient to further wander between different specialists and treatments.

Another explanation lies in the possibility that several of these diagnoses are incorrect, as stated in the first point. However, considering that we did not find a geographical prevalence, the problem of diagnostic classification and knowledge of these disorders is a common issue. On the other hand, it should be remembered that the first ICOP [1] classification is very recent, and its purpose is to provide clear and practical criteria for defining these syndromes in order to provide a common language, especially considering the various health operators involved in the diagnostic process of these subjects.

Therefore, it is not surprising that recent studies show the poor response of these patients to different pharmacological therapies and consultation with different specialists, often without real multidisciplinary teamwork [11,16,17].

Another point of interest raised from the data is that our sample reported receiving multiple diagnoses, with the final diagnosis often consisting of more than one. The sample was subdivided into four groups: isolated trigeminal neuralgia, trigeminal neuralgia, and other primary orofacial pain, two primary orofacial pain without trigeminal neuralgia, and a small group that continues to have no specific diagnosis. These results are not easy to understand due to the type of origin of the data, which is uncontrollable. However, they underline the possible important confusion of patients regarding the origin of their orofacial pain. Although it cannot be excluded that there are real coexistences of two primary forms of orofacial pain in the same patient, it cannot be denied that patients and doctors may have trouble evaluating this pain in a specific frame for each case.

In an attempt to better understand these data, each questionnaire was subjected to a group of researchers trained in the use of the two classifications (ICHD-3 and ICOP [1,9]) to see how to reallocate the diagnoses based on the answers to the questionnaires, obviously with all the limitations of post hoc control and without viewing health records. It is interesting to observe that more than 40% of trigeminal neuralgias change groups from idiopathic to symptomatic disorders, while tension headaches are reduced by more than 50% (which is reasonable), and both cluster headaches and migraines remain with a similar sample size. Furthermore, it is possible to attribute orofacial pain reported by a single patient to a single diagnosis. These findings may suggest that there is real confusion among patients and doctors in evaluating this pain in a specific frame for each case. Considering the insufficient results of treatments in more than 60% of the patients in our sample, both overall and for the different defined diagnosed groups, there are numerous primary headaches with involvement of the face that are treated inadequately. It cannot be excluded that several patients have been erroneously enrolled in the group of orofacial pains, despite having pains related to the topography of the skull. However, this appears less likely because the social groups for migraines and cluster headaches are much better known in Italy, as outpatient clinics for the treatment of headaches are considerably widespread. We surely need a population study to further investigate and support these results.

Finally, the sample confirmed the results of the literature [6,8] as subjects with orofacial pain frequently undergo visits by specialists of different specializations and receive multiple treatments, both pharmacological and non-pharmacological. However, the degree of dissatisfaction with both doctors and treatments remains high, with a reported high rate of side effects. About 50% had undergone neuroimaging.

Our data clearly suggest that an important update for health workers on these disorders is necessary to avoid troubled diagnostic procedures. The low degree of satisfaction found in our sample on the interventions carried out confirms these considerations. Research carried out on the general population and on the population belonging to a highly specialized clinic in these disorders would help to understand which are the greatest removable obstacles to effective treatment.

Furthermore, these data show how primary headaches in adulthood can have an orofacial localization, as already seen in pediatric ages [13,14], so this localization should not exclude headaches such as migraines or TACs from the differential diagnosis. There are several limitations to this study. The use of a questionnaire to collect data limits the ability to verify the accuracy of data obtained from medical records. The questionnaire method also implies the limitation of a retrospective analysis of collected data. Although methods for verifying the reliability of the answers were used, we cannot completely exclude the possibility that family members answered the questions instead of the patients. However, this seems unlikely, as subjects subscribed to a blog in which they actively participated and then asked others to answer the questionnaire. Further, some authors of GIMDO used questionnaires online with success [18]. Another limitation is that the sample may have been selected based on the severity of orofacial pain and failure or partial response to the treatments suggested by the different specialists. In fact, these blogs or associations often serve as help for pathologies that are difficult to treat or underestimated by public opinion (as in the case of painful syndromes). Nonetheless, analyzing our sample seems useful as it represents an unsatisfied clinical population with likely inaccurate and variable diagnoses. Furthermore, the geographic distribution of this study mirrors that of the general population. Another limitation of this study may be due to the lack of validation of the questionnaire, which is, however, primarily a survey of the diagnostic–therapeutic pathway that patients with orofacial non-odontogenic pain encounter. Understanding these difficulties may help to plan better strategies for patients. Furthermore, similar surveys have been used successfully by the authors.

Finally, our study’s generalizability may be limited as it is based on a particular population within a specific health system, which may make recognizing these disorders difficult. Finally, the generalizability of our study may be limited because it is based on a specific population within a specific health system, which may make identifying these conditions challenging. However, the literature demonstrates that the cited issues are shared by all Western countries [5].

Future research on clinical and general populations may help to establish a well-defined diagnostic and therapeutic pathway. Our data suggest the need for an integrated multidisciplinary intervention among different specialists, avoiding isolated therapeutic diagnostic attempts but facilitating communication between different specialists. Our multidisciplinary group’s next step will be to propose an integrated therapeutic diagnostic pathway and validate it on clinical populations.

## 5. Conclusions

The findings of this study, such as the high prevalence of primary migraine and tension headaches and the low prevalence of persistent idiopathic facial headache, have important implications for the diagnosis and treatment of orofacial pain. Clinicians should be aware of these patterns and use them to guide their diagnostic and treatment decisions. For example, patients with primary migraine or tension headaches may benefit from medications such as triptans and nonsteroidal anti-inflammatory drugs, while those with persistent idiopathic facial headache may escape to the medical diagnosis, while these disorders require instead more specialized treatment approaches.

## Figures and Tables

**Table 1:** Orofacial pain GIDMO survey: demographic characteristics.

Dates		Numbers %	
Participants		320	100.0
Gender	Male	76	23.7
	Female	244	76.3
Age (years)	18–35	55	17.2
	36–55	176	55.0
	56+	89	27.8
Geographic area	North Italy	139	43.4
	Central Italy + Sardinia	66	20.7
	South Italy + Sicily	112	35.0
	Foreign countries	3	0.9

**Table 2:** Orofacial pain GIDMO survey: referred diagnosis.

Dates		Numbers	%
Diagnosis numbers			
	None	12	3.7
	One	192	60.0
	Two or more	116	36.3
Diagnosis type			
	Trigeminal neuralgia	178	41.1
	Cluster headache	58	13.4
	Migraine with or without aura	47	10.8
	Other neuralgia	44	10.2
	Tension headaches	35	8.3
	Temporomandibular joint dysfunction	27	6.2
	Dental pathologies	20	4.6
	Sinusitis	15	3.5
	Herpes zoster, neuralgia psot-HZV	8	1.8
	Cranial nerve irritations	0	0.0
	Musculoskeletal disorders	0	0.0
	Glaucoma or other ophthalmopathy	0	0.0
	Others	0	0
	None	12	3.8
	Total	433	100.0

**Table 3:** Orofacial pain GIDMO survey: temporal characteristics of pain.

Questions		Numbers	%
When did the pain begin?			
	Less than 3 months	10	3.1
	More than 3 months but less than 1 year	16	5.0
	From one to three years	44	13.8
	More than 3 years	250	78.1
Did the pain spontaneously disappear after its beginning?			
	Yes	105	32.8
	No	215	67.2
If yes, did the pain come back again after some time?			
	Yes	95	90.5
	No	8	7.6
	Missing data	2	1.9
Was your pain always present during the day even if with different intensity?			
	Yes	201	62.8
	No	119	37.2
The pain crisis manifested			
	as a single attack	121	37.8
	as series of attacks	199	62.2
Pain only manifests with crises of limited duration			
	Yes	131	40.9
	No	189	59.1
If yes, how long do these crises last?			
	From a few seconds to less than 2 min	38	29.0
	From 2 to 10 min	18	13.7
	More than 10 min	73	55.7
	Does not answer	2	1.5
Did pain crises reach their peak in a very short time and disappear immediately?			
	Yes	80	25.0
	No	240	75.0
Did any underlying pain persist in the same area where pain crises occur?			
	Yes	254	79.4
	No	66	20.6
Pain is felt			
	on one side of the face	269	84.1
	on both sides	51	15.9

**Table 4:** Orofacial pain GIDMO survey: peculiar pain feature.

Questions		Numbers	%
Did the pain tend to reduce and/or disappear after standing the face motionless for a few minutes?			
	Yes	95	29.7
	No	225	70.3
Was the pain also felt in the back of the head, ears, neck or throat?			
	Yes	209	6.3
	No	111	34.7
Was the pain caused by harmless or non-painful stimuli (such as touching face, shaving, talking, laughing, brushing teeth, applying make-up, chewing, a light breeze, etc.)?			
	Yes	197	61.6
	No	123	37.4
Did the eye from the pain side get red or watery during pain crises?			
	Yes	174	54.4
	No	146	45.6
Did the side of the nose from the pain side get blocked during pain crises?			
	Yes	145	45.3
	No	175	54.7
Did the face from the side of the pain sweat during pain crisis?			
	Yes	63	19.7
	No	257	80.3
Did the upper eyelid on the pain side droop during pain crises?			
	Yes	206	64.4
	No	114	35.6
Was the onset of pain preceded by a facial trauma/surgery on the face or mouth/radiation treatment in the previous 3 months?			
	Yes	42	13.1
	No	278	86.9
Was the onset of pain preceded, in the previous 3 months, by dental treatment?			
	Yes	82	25.6
	No	238	74.4
Was the onset of pain preceded in the previous month by a herpetic infection?			
	Yes	12	3.7
	No	308	96.3

**Table 5:** (**a**). Orofacial pain GIDMO survey: diagnostic approach. (**b**). Orofacial pain GIDMO survey: diagnostic approach. Continuation.

(**a**)			
**Questions**		**Numbers**	**%**
Which doctor did you go to first when the pain began?			
	Neurologist	90	28.1
	Dentist	84	26.3
	General practitioner	70	21.9
	Emergency doctor	24	7.5
	Otolaryngologist	14	4.4
	Neurosurgeon	10	3.1
	Pain doctor	8	2.5
	Other	8	2.5
	Oculist	6	1.9
	Maxillofacial surgeon	6	1.9
How many investigations were prescribed first? (more than one answer possible)			
	None	11	3,4
	One	102	31.9
	More than one	207	64.7
Which investigations were prescribed first? (more than one answer possible)			
	MRI brain	187	34.4
	Dental X-ray/CT scan	136	25.0
	Brain CT scan	87	16.0
	MRI/Ct facial mass and/or temporomandibular joint	71	13.1
	X-ray of the head and/or of the cervical spine	47	8.7
	Neurophysiological examines, such as blink-reflex	15	2.8
	Total	543	100.0
After the first visit, have you had an official diagnosis?			
	Yes	218	68.1
	No	102	31.9
If yes, how many? (more than one answer possible)			
	Missing data	2	3.4
	One	156	71.5
	More than one	60	27.5
If yes, which one? (more than one answer possible)			
	Trigeminal neuralgia	119	46.9
	Cluster headache	30	11.6
	Tension headache	25	9.6
	Temporomandibular joints dysfunction	20	8.1
	Sinusitis	13	7.7
	Dental pathologies	13	7.7
	Not-trigeminal neuralgia.	12	4.6
	Irritations of other cranial nerves	10	3.9
	Others	9	3.5
	Musculoskeletal disorders	3	1.2
	Glaucoma or other ophthalmopathy	3	1.2
	Herpes zoster, post-herpetic neuralgia	2	0.8
	Total	259	100.0
Was the diagnosis changed in a second time?			
	Yes	103	32.2
	No	182	56.9
	No diagnosis made yet	35	10.9
(**b**)			
**Questions**		**Numbers**	**%**
How many times was the diagnosis changed before getting the current one?			
	Never	167	52.2
	One–two times	71	22.2
	Three–four times	33	10.3
	More than five times	15	4.7
	I have no specific diagnosis	34	10.6
Were you referred to another specialist after the first visit?			
	Yes	227	70.9
	No	93	29.1
If yes, how many specialists?			
	One specialist	151	66.6
	Two or more specialists	75	33.0
	Does not answer	1	0.4
If yes, which specialist? (more than one answer possible)			
	Missing data	2	3.4
	One	156	71.5
	More than one	60	27.5
If yes, which specialist? (more than one answer possible)			
	Neurologist	134	34.0
	Dentist	32	7.9
	General practitioner	16	3.9
	Emergency doctor	6	1.9
	Otolaryngologist	30	7.4
	Neurosurgeon	73	18.0
	Pain doctor	40	9.8
	Others	8	2.0
	Oculist	21	5.3
	Maxillofacial surgeon	46	11.3
	Total	406	100.0

**Table 6:** Orofacial pain GIDMO survey: drug treatment.

Questions		Numbers	%
Has drug therapy been prescribed? (more than one answer possible)			
	Yes	307	95.9
	No, never	13	4.1
If yes, indicate how many drugs were taken (more than one answer possible)			
	One	112	35.2
	Two–three	123	38.6
	Four–five	43	13.6
	Six–seven	24	7.5
	Eight–nine	16	5.0
If yes, indicate which drug(s) was taken (more than one answer possible)			
	Anticonvulsant	226	37.0
	FANS	112	14.9
	SSRI	91	12.1
	Opioid	90	12.0
	Anxiolytic	57	7.6
	Others	54	7.2
	muscle relaxant	50	6.6
	Paracetamol	45	6.0
	Cannabis	27	3.6
	Total	752	100.0
If yes, was it effective?			
	Yes	174	54.4
	No	127	39.7
	Missing data	19	5.9

**Table 7:** Orofacial pain GIDMO survey: non-pharmacological treatment.

Questions		Numbers	%
Was a non-pharmacological treatment recommended?			
	Yes	163	50.8
	No	157	49.1
If yes, when?			
	Does not respond	6	3.7
	After drug treatment failure	63	38.6
	Along with drug treatment	79	48.5
	Before drug treatment	7	4.3
	Never	8	4.9
If yes, how many non-pharmacological treatments have been prescribed?			
	One	100	65.8
	Two–three	40	26.3
	Four–eight	12	7.9
If yes, which non-pharmacological treatment was prescribed? (more than one answer possible)			
	Microvascular decompression	48	21.0
	Others	46	20.2
	Local injections with or without radiological or ultrasound guide (for example, botulinic toxin or anesthetic)	35	15.3
	Radiofrequency thermorhizotomy	34	14.9
	Gamma knife treatment	22	9.6
	Alcohol injection or glycerol in Gasser ganglia	18	7.9
	Balloon micro compression	15	6.6
	Electricity treatment (for example PENS)	10	4.4
	Acupuncture	10	4.4
	Osteopathy	4	
	Total	228	100.0

**Table 8:** Orofacial pain GIDMO survey: final questions.

Questions	No.	Median	Min. Max
Pain severity			
Indicate the pain grade assigned to the crisis or to the series of pain crises	320	10	0–10
Indicate the grade assigned to the background pain (if any)	254	7	0–10
Level of satisfaction of			
The first diagnosis	320	5	0–10
Drugs therapy	307	5	0–10
Discomfort regarding any side effects that may have occurred	301	8	0–10
Indicate the level of satisfaction with the first invasive treatment (if any)	111	5	0–10
Indicate the level of discomfort regarding any side effects of the invasive treatment (if any)	112	5	0–10

## Data Availability

Data are in possess of the authors in the format excel.

## References

[1] The Orofacial Pain Classification Committee (2020). International Classification of Orofacial Pain, 1st Edition (ICOP). Cephalalgia.

[2] Ziegeler C., May A. (2020). The ICHD Definition of “facial Pain” Should Be Revised. Cephalalgia.

[3] Zakrzewska J.M. (2013). Differential Diagnosis of Facial Pain and Guidelines for Management. Br. J. Anaesth..

[4] Ziegeler C., May A. (2019). Facial Presentations of Migraine, TACs, and Other Paroxysmal Facial Pain Syndromes. Neurology.

[5] Ziegeler C., Beikler T., Gosau M., May A. (2021). Idiopathic Facial Pain Syndromes—An Overview and Clinical Implications. Dtsch. Arztebl. Int..

[6] May A., Hoffmann J. (2021). Facial Pain beyond Trigeminal Neuralgia. Curr. Opin. Neurol..

[7] Siccoli M.M., Bassetti C.L., Sándor P.S. (2006). Facial Pain: Clinical Differential Diagnosis. Lancet Neurol..

[8] Peng K.P., Benoliel R., May A. (2022). A Review of Current Perspectives on Facial Presentations of Primary Headaches. J. Pain. Res..

[9] Olesen J. (2018). Headache Classification Committee of the International Headache Society (IHS) The International Classification of Headache Disorders, 3rd Edition. Cephalalgia.

[10] Zebenholzer K., Wöber C., Vigl M., Wessely P., Wöber-Bingöl Ç. (2005). Facial Pain in a Neurological Tertiary Care Centre--Evaluation of the International Classification of Headache Disorders. Cephalalgia.

[11] Ziegeler C., Brauns G., May A. (2021). Characteristics and Natural Disease History of Persistent Idiopathic Facial Pain, Trigeminal Neuralgia, and Neuropathic Facial Pain. Headache.

[12] Yoon M.S., Mueller D., Hansen N., Poitz F., Slomke M., Dommes P., Diener H.C., Katsarava Z., Obermann M. (2010). Prevalence of Facial Pain in Migraine: A Population-Based Study. Cephalalgia.

[13] Correnti E., Lo Cascio S., Cernigliaro F., Rossi R., D’Agnano D., Grasso G., Pellegrino A., Lauria B., Santangelo A., Santangelo G. (2023). Idiopathic Non-Dental Facial Pain Syndromes in Italian Children: A Clinical Case Series. Life.

[14] Raieli V., Reina F., D’agnano D., Nocera G.M., Capizzi M., Marchese F., Sciruicchio V. (2022). The Pediatric Trochlear Migraine: Diagnostic and Therapeutic Implications. J. Clin. Med..

[15] Crandall J.A. (2018). An Introduction to Orofacial Pain. Dent. Clin. N. Am..

[16] Sharav Y., Haviv Y., Benoliel R. (2023). Orofacial Migraine or Neurovascular Orofacial Pain from Pathogenesis to Treatment. Int. J. Mol. Sci..

[17] Clark G.T., Dionne R.A. (2012). Orofacial Pain: A Guide to Medications and Management.

[18] Manzo M.L., Reina F., Correnti E., D’Aiuto F., D’Agnano D., Santangelo A., Vetri L., Santangelo G., Maniscalco L., Tripi G. (2023). Evolution of Pediatric Migraine Patients Admitted at an Emergency Department after a 10-Year Follow-Up. J. Clin. Med..

